# High Thermoelectric Performance in 2D Sb_2_Te_3_ and Bi_2_Te_3_ Nanoplate Composites Enabled by Energy Carrier Filtering and Low Thermal Conductivity

**DOI:** 10.1021/acsaelm.3c00385

**Published:** 2023-06-05

**Authors:** Tanner Q. Kimberly, Kamil M. Ciesielski, Xiao Qi, Eric S. Toberer, Susan M. Kauzlarich

**Affiliations:** † Department of Chemistry, University of California, One Shields Avenue, Davis, California 95616, United States; ‡ Department of Physics, Colorado School of Mines, 1523 Illinois Street, Golden, Colorado 80401, United States; § The Molecular Foundry, Lawrence Berkeley National Lab, Berkeley, California 94720, United States

**Keywords:** nanocomposites, colloidal synthesis, Bi_2_Te_3_, Sb_2_Te_3_, thermoelectrics, zT > 1, energy carrier filtering

## Abstract

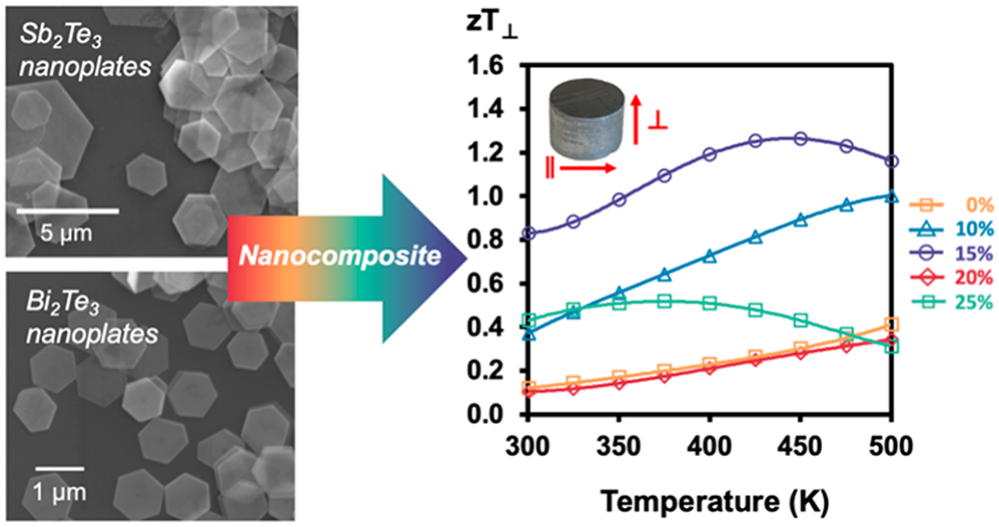

Thermoelectrics are an important class of materials with great potential in alternative energy applications. In this study, two-dimensional (2D) nanoplates of the layered chalcogenides, Sb_2_Te_3_ and Bi_2_Te_3_, are synthesized and composites of the two are investigated for their thermoelectric properties. The two materials, Sb_2_Te_3_ and Bi_2_Te_3_, were synthesized as hexagonal, 2D nanoplates via a colloidal polyol route. The as-synthesized Sb_2_Te_3_ and Bi_2_Te_3_ vary drastically from one another in their lateral and vertical dimensions as revealed by scanning electron microscopy and atomic force microscopy. The single crystalline nanoplate nature is deduced by high-resolution transmission electron microscopy and selected area electron diffraction. Nanoplates have well-defined hexagonal facets as seen in the scanning and transmission electron microscopy images. The nanoplates were consolidated as an anisotropic nanostructured pellet via spark plasma sintering. Preferred orientation observed in the powder X-ray diffraction pattern and scanning electron microscopy images of the fractured pellets confirm the anisotropic structure of the nanoplates. Thermoelectric properties in the parallel and perpendicular directions were measured, revealing strong anisotropy with a significant reduction to thermal conductivity in the perpendicular direction due to increased phonon scattering at nanoplate interfaces. All compositions, except that of the 25% Bi_2_Te_3_ nanoplate composite, behave as degenerate semiconductors with increasing electrical resistivity as the temperature increases. The Seebeck coefficient is also increased dramatically in the nanocomposites, the highest reaching 210 μV/K for 15% Bi_2_Te_3_. The increase in Seebeck is attributed to energy carrier filtering at the nanoplate interfaces. Overall, these enhanced thermoelectric properties lead to a drastic increase in the thermoelectric performance in the perpendicular direction, with *zT* ∼ 1.26, for the 15% Bi_2_Te_3_ nanoplate composite at 450 K.

## 1 Introduction

With the current infrastructure of the 21st century, approximately two-thirds of the total energy consumed is lost as rejected energy in the form of heat.^1^ Therefore, the ability to sequester heat and convert it into usable energy is extremely desirable and can be a viable renewable energy source. One such way to accomplish this task is to design and implement efficient thermoelectric materials, which can convert heat into electricity. The efficiency of a thermoelectric material is described by the dimensionless figure of merit *zT*, which is given by *zT* = (*S*^2^*T*)/*ρκ*.

The *zT* value is dictated by the Seebeck coefficient (*S*), electrical resistivity (ρ), and thermal conductivity (κ) of the material. Many thermoelectric materials have either low κ, low ρ, or high *S*, but it is difficult to optimize all these parameters simultaneously as they are inversely related through carrier concentration.^2^

Thermoelectric materials, under optimized conditions, have great potential for power generation when implemented in solid state thermoelectric generators.^3^ Over the past decades, there have been many strategies implemented to improve the efficiency of thermoelectric materials. Such strategies include electronic band convergence,^4^ nanostructuring,^5^ and energy carrier filtering effects.^6^ With the advancement of nanostructuring, the field of thermoelectrics has come closer to the realization of a phonon-glass electron-crystal system, through the reduction in lattice thermal conductivity (*κ*_l_) and increase in density-of-states that nanomaterials offer.^7^ The observation of energy carrier filtering in multiphase materials, arising from the potential barrier that the charge carriers face when transporting across interfaces has further advanced thermoelectric research.^8^ Previous studies report high thermoelectric figures of merit for Bi_2–*x*_Sb_*x*_Te_3_/SiC^9^ and Bi_2_Te_3_/Yb_2_O_3_^10^ nanostructured composites, attributed to high Seebeck coefficients arising from an energy carrier filtering mechanism, and reduced *κ*_l_ from increased phonon scattering interfaces. Energy carrier filtering has also led to the realization of enhanced thermoelectric performance in a variety of other composites including Mg_3_Sb_2_ with graphene nanoplatelets,^11^ Ag_2_Se with poly(3,4-ethylenedioxythiophene) (PEDOT),^12^ and Ag_2_Se with carbon nanotubes.^13^

The materials Sb_2_Te_3_ and Bi_2_Te_3_ are well-known thermoelectrics that have peak *zT* values of around 1.0 at room temperature with optimization, competitive with many currently employed room-temperature thermoelectric materials.^14^ Alloys of Sb_2_Te_3_, with Bi_2_Te_3_ and Bi_2_Se_3_, were discovered in the 1960s, and are still among the highest performing room-temperature thermoelectric materials to-date.^15^ The thermoelectric properties of Sb_2_Te_3_ and Bi_2_Te_3_ have been investigated over the years and their high performance is largely attributed to their high band degeneracy, low electrical resistivity, and thermal conductivity.^16^ It is predicted that the lattice thermal conductivity, *κ*_l_, can be decreased further in these materials by nanoscale engineering due to the increase in phonon scattering sites.^17^ The thermoelectric performance can be electronically modulated by introducing p–n junctions in bulk Sb_2_Te_3_ and Bi_2_Te_3_ which has been shown to enhance Seebeck values through energy carrier filtering.^18^ The practical applications of Bi_2_Te_3_ and its alloys in thermoelectric generators have been investigated and their thermal and mechanical properties are well-known.^19^ Bi_2_Te_3_-based materials find applications in niche areas of thermoelectric power generation, such as in solar thermoelectric generators^20^ and wearable electronics.^21^

There have been recent reports on solution synthesis of two-dimensional (2D) thermoelectric nanomaterials, thus enabling control over their thermal conductivity and electronic properties.^22−24^ The most notable advances in solution route syntheses have been with hydrazine-assisted, surfactant-assisted, and polyol colloidal synthetic procedures, enabling the realization of 2D chalcogenide nanoplates at low-temperatures and under ambient conditions.^22,25^ These 2D nanostructures have shown significant enhancements in Seebeck coefficients as well as lowered *κ*_l_.^24,26^ There are many examples of solution route synthesis and thermoelectric properties of 2D nanoplates of Sb_2_Te_3_^27−29^ and Bi_2_Te_3_^30,31^ that exhibit lowered κ with respect to the bulk.^32,33^ Recent examples of Bi_2_Te_3_ and Bi_2_Se_3_ nanoflake composites show low κ values ranging from 0.55 to 0.68 W m^–1^ K^–1^, as well as enhanced *S* of ∼220 μV/K enabled by energy carrier filtering.^34^ Nanoflower composites of Sb_2_Te_3_ and Bi_2_Te_3_ have also shown improvements in the thermoelectric efficiency due to increased Seebeck coefficients.^35^ Increased thermoelectric figure of merit has been observed in Bi_2_Te_3_/Sb_2_Te_3_ core–shell heterostructure nanoplates that arise from a high *S* of 145 μV/K.^36^

Inspired by these studies, 2D nanoplates of Sb_2_Te_3_ and Bi_2_Te_3_ were synthesized and pressed into dense pellets keeping their anisotropic structure orientation. The thermoelectric properties of the materials and composites of the two were measured. The composite provides a simple approach to modulate the electronic transport, through morphology control and composition. The resistivity and Seebeck coefficients were studied as a function of direction and nanoplate composition. Furthermore, we report successful control over thermal conductivity by exploiting the anisotropic structure and varying the composition of the nanoplates. 2D Sb_2_Te_3_ and Bi_2_Te_3_ nanoplates were colloidally synthesized via a polyol method. The as-synthesized nanoplates were characterized by powder X-ray diffraction (PXRD), scanning electron microscopy (SEM), transmission electron microscopy (TEM), selected area electron diffraction (SAED), and atomic force microscopy (AFM). The nanoplates were consolidated into a high-density pellet via spark plasma sintering (SPS) and the pellet was further characterized by PXRD, SEM and energy dispersive X-ray spectroscopy (EDX). The thermoelectric properties of the nanoplate pellets were measured, exhibiting enhanced thermoelectric performance in the temperature range from 300 to 500 K.

## 2 Experimental Section

### 2.1 Synthesis of Nanoplates

Colloidal synthesis of the Sb_2_Te_3_ nanoplates was performed by stirring 3.283 g of SbCl_3_ (Sigma-Aldrich, 99.95%), which was weighed inside of a glovebox to exclude moisture, 3.990 g of Na_2_TeO_3_ (Sigma-Aldrich, 99%), and 2.700 g of polyvinylpyrrolidone (PVP) (Sigma-Aldrich, ∼55 000 MW) in 200 mL of a 0.5 M sodium hydroxide (Fisher Scientific) and diethylene glycol (Sigma-Aldrich, 99%) solution. The sodium hydroxide in diethylene glycol solution was dried over 3 Å molecular sieves, at ∼20 vol %, for at least 24 h prior to use. The reaction mixture was degassed and purged with argon gas three times. The reaction was accomplished in a 1 L three-neck round-bottom flask equipped with a heating mantle and condenser at 210 °C for 18 h under argon gas flow. Temperature was controlled with a thermocouple placed directly into the reaction solution. After heating, the reaction solution was allowed to cool to room temperature naturally and then evenly aliquoted into eight 50 mL centrifuge tubes. Subsequently, 25 mL of acetone was added to each solution. The nanoplates were subjected to centrifugation at 8500 rpm for 5 min. The dark supernatant was discarded, and the pellet was redispersed in 30 mL of ethanol. The nanoplates were washed two more times with ethanol, and then three more times with water. Finally, the nanoplates were dispersed in ethanol.

Colloidal synthesis of the Bi_2_Te_3_ nanoplates was accomplished by stirring 5.821 g of Bi(NO_3_)_3_·5H_2_O (Alfa Aesar, 98%), 3.990 g of Na_2_TeO_3_ (Sigma-Aldrich, 99%), and 4.000 g of polyvinylpyrrolidone (PVP) (Sigma-Aldrich, ∼ 40 000 MW) in 200 mL of a 0.375 M sodium hydroxide (Fisher Scientific) and ethylene glycol (Sigma-Aldrich, 99%) solution. The sodium hydroxide in ethylene glycol solution was dried over 3 Å molecular sieves, at ∼20 vol %, for at least 24 h prior to use. The reaction mixture was degassed and purged with argon gas three times. The reaction was run in a 1L three-neck round-bottom flask equipped with a heating mantle and condenser at 185 °C for 5 h under argon gas flow. Temperature was controlled with a thermocouple placed directly into the reaction solution. After heating, the reaction solution is allowed to cool to room temperature naturally and the product was washed the same way as previously described.

Once isolated, the nanoplates were precipitated by centrifugation and dried for 2 h under vacuum to remove the residual solvent. The dried ingot was ground with an agate mortar and pestle, and the powder was sieved. To make the nanoplate composite, the separate Sb_2_Te_3_ and Bi_2_Te_3_ nanoplate powders were weighed according to specific mole percentages and stirred in 100 mL of ethanol overnight. Once mixed, the nanoplates were again precipitated by centrifugation, dried for 2 h under vacuum, and the ingot was annealed for 1 h at 300 °C in an alumina ceramic crucible boat in a tube furnace under argon gas flow to thoroughly remove any remaining surfactant, as previously described by Liu et al.^37^

### 2.2 Characterization of Nanoplates

The nanoplates were analyzed by powder X-ray diffraction (PXRD) on a Bruker D8 Advance diffractometer using Cu Kα radiation operated at 40 kV and 25 mA at room temperature. The size and morphology of the nanoplates were assessed using a Thermo Fisher Quattro S Environmental scanning electron microscope (SEM) operated at 15 kV and JEOL 2100F transmission electron microscope (TEM) operated at 200 kV. High-resolution TEM (HRTEM) and selected-area electron diffraction (SAED) were acquired using a FEI ThemIS 60–300 STEM/TEM (Thermo Fisher Scientific, US) operated at 300 kV at the National Center for Electron Microscopy within the Molecular Foundry in Lawrence Berkeley National Laboratory. The ThemIS is equipped with image aberration corrector optics, and a Ceta2 camera (4k × 4k pixels, and 14-bit dynamic range). The thickness of the nanoplates was measured using an Asylum MFP-3D atomic force microscope (AFM) operated in tapping mode.

### 2.3 Spark Plasma Sintering

The thermoelectric properties were measured on the consolidated powder in the form of a pellet, prepared using a Dr. Sinter Junior Spark Plasma Sintering SPS-2ll LX system (Fuji Electronic Industrial Co., LTD) under vacuum. The nanoplate powders (∼3–4 g) were loaded into a 10 mm graphite die with graphite plungers and 16 pieces of graphite foil on each side to ensure air-free conditions. The 10 mm die was inserted into a larger 20 mm die with graphite plungers and 6 graphite foils on each side, with a thermocouple placed into a hole drilled into the 20 mm die to ensure precise temperature control. The nanoplate powder was initially cold pressed at 45 MPa for 3 min under static vacuum. After the cold press, the holding pressure was increased to 89 MPa over 5 min and held for the remaining 10 min, while the die was simultaneously heated from room temperature to 370 °C over 3 min and then to 400 °C over 1 min and held at 400 °C for 11 min. The die was naturally cooled after the SPS process and an ∼8 mm thick gray/metallic pellet was obtained. The pellet was cut in two directions, parallel and perpendicular to the SPS direction, and polished to obtain one 10 mm diameter by 1 mm thick circular pellet and a 6 mm^2^ by 1 mm thick square pellet. The circular and square pellets were also analyzed by PXRD and SEM to obtain information about the preferred orientation and composition of the nanoplate pellet. The pellets were further analyzed by EDX to probe the composition and elemental dispersion of the two nanoplates phases within the pellet.

### 2.4 Thermoelectric Property Measurements

Thermal diffusivity of the sample was measured using a Netzsch Laser Flash Analysis (LFA) instrument, from which κ was calculated, using the equation κ(*T*) = *ρC*_p_*α*(*T*), where ρ is the density, *C*_p_ is the heat capacity, and α(*T*) is the thermal diffusivity. Electronic measurements were carried out on the sample to determine carrier transport. Temperature dependent *S* was measured on a custom apparatus under high vacuum from 300 to 500 K.^38^ In addition, both the Hall effect and ρ were measured on a custom-built apparatus in the same temperature range and vacuum pressure as that for *S* measurement.^39^ The four-probe van der Pauw method was used to deduce Hall and conductivity. All electronic transport measurements were performed with heating and cooling cycles to show that samples do not evolve with temperature cycling.

## 3 Results and Discussion

### 3.1 Nanoplate Structure and Morphology Analysis

**Figure 1:**
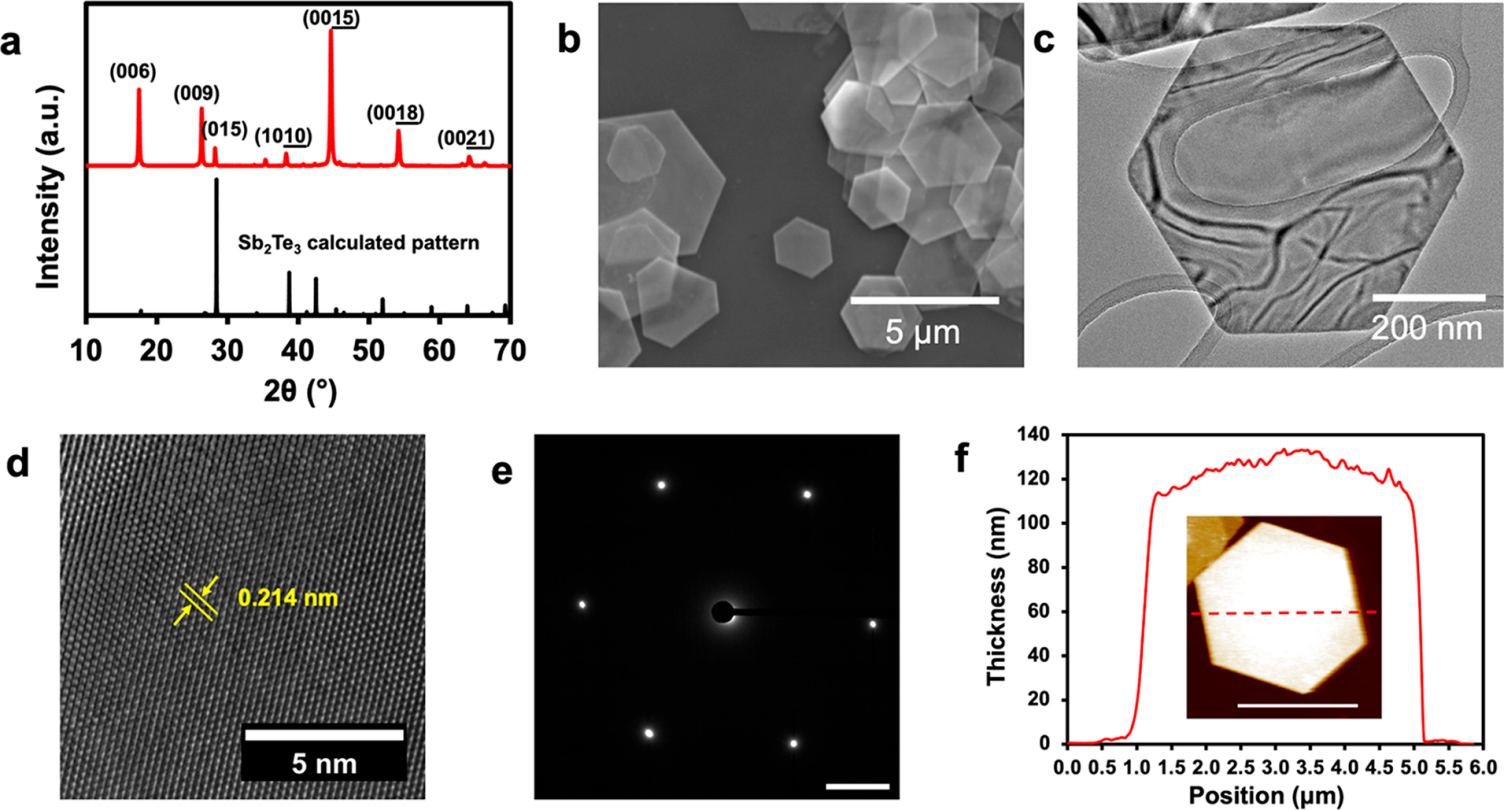
Sb_2_Te_3_ nanoplates characterized with (a) PXRD pattern, (b) SEM micrograph, (c) TEM micrograph, (d) HRTEM image showing a measured lattice spacing of 0.214 nm for the (1120) plane, (e) SAED pattern (scale bar 2 nm^–1^), and (f) AFM measured thickness of ∼130 nm, with AFM image shown in the inset (scale bar 3 μm).

The Sb_2_Te_3_ and Bi_2_Te_3_ nanoplate reactions were performed on a 200 mL scale with a yield of ∼90%, which produces ∼3.4 g of Sb_2_Te_3_ and ∼4.3 g of Bi_2_Te_3_. As-synthesized Sb_2_Te_3_ nanoplates are phase-pure by PXRD and the diffraction peaks are indexed to the rhombohedral Sb_2_Te_3_ crystal structure as shown in Figure 1a. It is important to note that if the sodium hydroxide solution is not dried with molecular sieves, then elemental Te is often present as a side product (Supporting Information, Figure S1). Preferred orientation of the nanoplates on the PXRD substrate is observed by the enhancement of the (00*l*) Miller indices. The nanoplates display a hexagonal morphology and a relatively large distribution in their lateral dimension of 2–7 μm, as shown in the SEM micrograph of Figure 1b. A typical Sb_2_Te_3_ nanoplate with hexagonal morphology and sharp facets is shown in the TEM micrograph of Figure 1c, where shiny fringes are observed from electron interference with the 2D nanoplate. The crystallinity of a single Sb_2_Te_3_ nanoplate is also investigated using high-resolution transmission electron microscopy (HRTEM) and selected area electron diffraction (SAED). The lattice fringes of a typical Sb_2_Te_3_ nanoplate are shown in the HRTEM image of Figure 1d. The (1120) lattice plane is highlighted, and the corresponding lattice spacing is measured to be 0.214 nm, agreeing excellently with the known crystal structure.^40^Figure 1e is the nanoplate SAED pattern, showing six distinct diffraction spots along the [0001] zone axis, all corresponding to a specific Miller index. The six sharp diffraction spots of the SAED pattern indicate that the nanoplates are single-crystalline nanoplates. The AFM measurements, as shown in Figure 1f, reveal that the thickness of the Sb_2_Te_3_ nanoplates varies between nanoplates, reaching a maximum thickness of ∼130 nm as shown in the AFM image. The AFM measurement reveals a dome-like surface of the Sb_2_Te_3_ nanoplates, which could be due to a screw dislocation driven growth that has been observed previously in this material^41^ as well as other 2D chalcogenides.^42^

**Figure 2:**
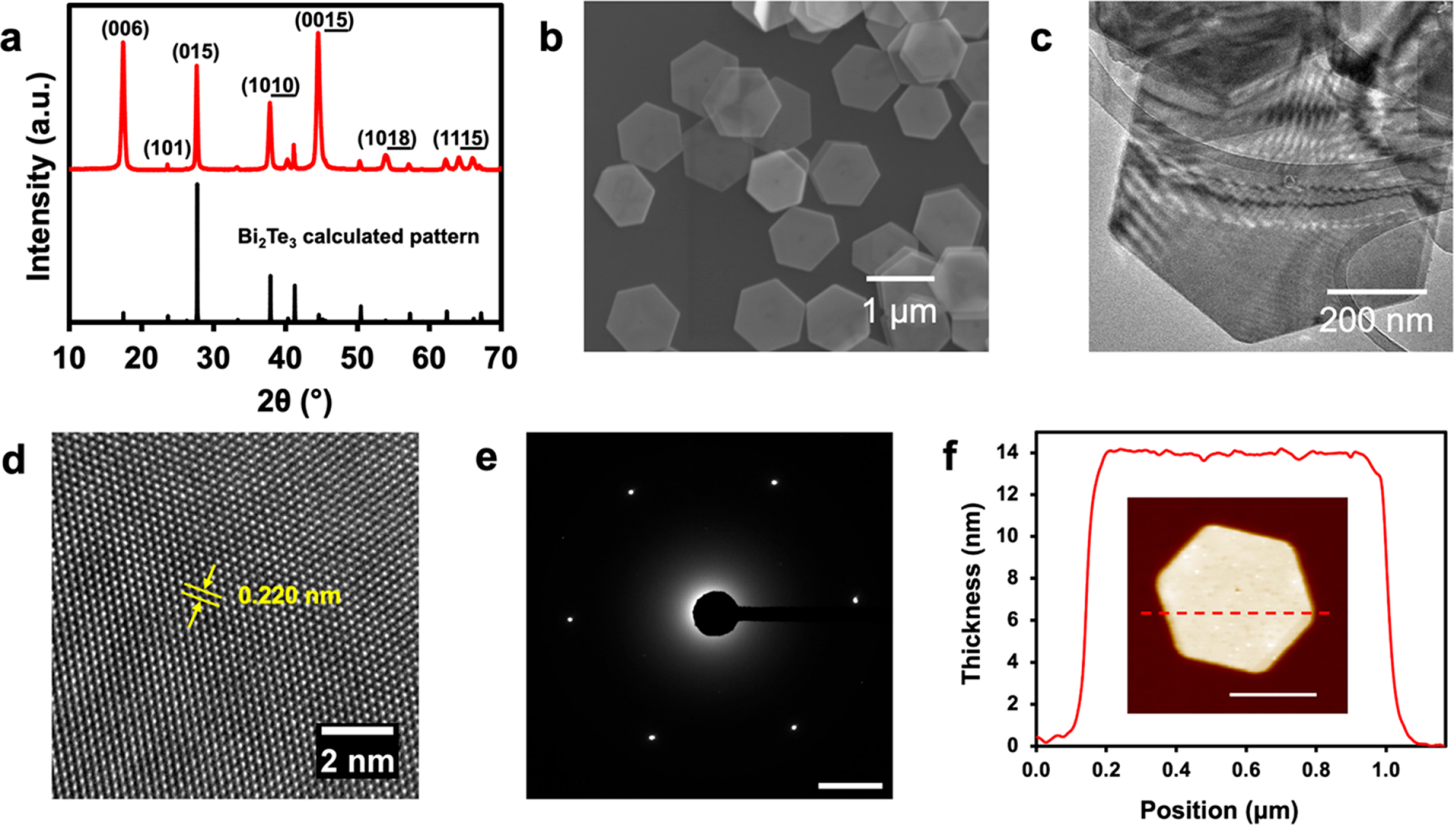
Bi_2_Te_3_ nanoplates characterized with (a) PXRD pattern, (b) SEM micrograph, (c) TEM micrograph, (d) HRTEM image showing a measured lattice spacing of 0.220 nm for the (1120) plane, (e) SAED pattern (scale bar 2 nm^–1^), and (f) AFM measured thickness of ∼14 nm, with an AFM image shown in the inset (scale bar 0.5 μm).

As-synthesized Bi_2_Te_3_ nanoplates are phase-pure by PXRD, and the diffraction peaks are indexed to the rhombohedral Bi_2_Te_3_ crystal structure as shown in Figure 2a. If the sodium hydroxide solution is not dried with molecular sieves, then elemental Te is often present as a side product or nanoplates form a single nanopore in the center (Supporting Information, Figure S2). Preferred orientation of the nanoplates can be observed in the diffraction pattern. The nanoplates have a hexagonal morphology but range from 0.5 to 1 μm in diameter as shown in Figure 2b. The Bi_2_Te_3_ nanoplates have a narrower distribution in their lateral dimension than the Sb_2_Te_3_ nanoplates. A typical Bi_2_Te_3_ nanoplate is shown in the TEM micrograph of Figure 2c, displaying the hexagonal morphology and sharp facets of the nanoplate. Electron interference fringes are observed in the TEM image. The lattice fringes of a typical Bi_2_Te_3_ nanoplate are shown in the HRTEM image of Figure 2d, where the lattice spacing for the (1120) plane is 0.220 nm, which is in excellent agreement with the previously reported crystal structure.^43^ The SAED pattern is shown in Figure 2e, displaying six distinct diffraction spots which correspond to specific Miller indices when imaged down the [0001] zone axis. The sharp and distinct diffraction spots, in addition to the highly ordered lattice fringes in the HRTEM image, indicate the single crystal nature of the Bi_2_Te_3_ nanoplates. The measured thickness of a typical Bi_2_Te_3_ nanoplate by AFM is ∼14 nm, as shown in Figure 2f.

### 3.2 Characterization of Consolidated Nanoplate Pellets

Due to the anisotropy of the layered Sb_2_Te_3_ and Bi_2_Te_3_ crystal structures, thermoelectric properties are also anisotropic.^44^ In order to elucidate the anisotropy in thermoelectric performance of the nanoplates, the consolidated nanoplate pellets are cut in two different directions denoted “parallel” and “perpendicular”. In the parallel direction, the thermoelectric property measurements are performed parallel to the nanoplate plane. In the perpendicular direction, thermoelectric properties are measured perpendicular to the nanoplate plane. For all composites, Sb_2_Te_3_ is the majority phase and Bi_2_Te_3_ is added in relatively small amounts (0 to 25 mol %). Therefore, they will be described according to the % of Bi_2_Te_3_ in the composite: for example, 25% refers to the 75% Sb_2_Te_3_/ 25% Bi_2_Te_3_ nanoplate composite. All pellets were 90 ± 2% of their theoretical density, determined by the Archimedes principle (Supporting Information, Figure S3).

**Figure 3:**
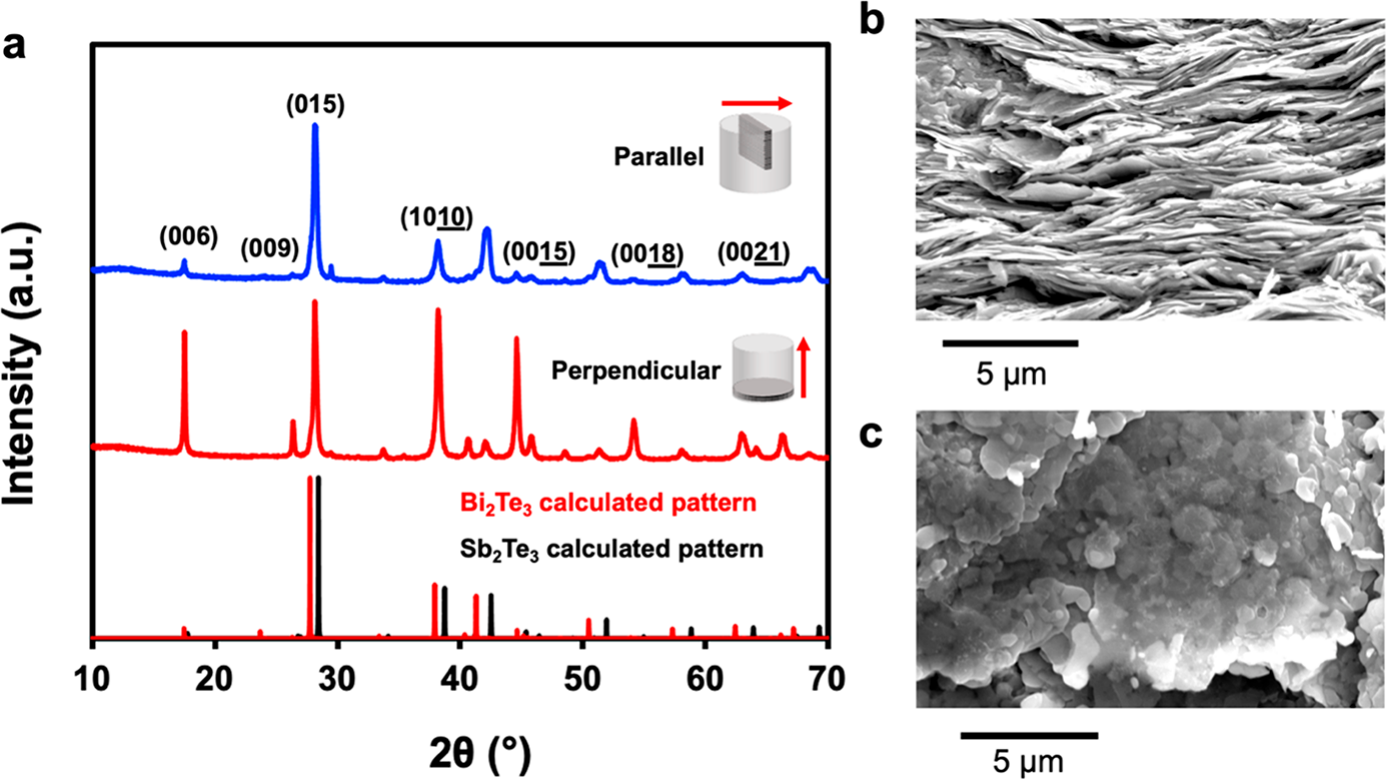
(a) PXRD pattern of the 25% nanoplate composite in the parallel and perpendicular directions and SEM micrographs of nanoplate composite in the (b) parallel and (c) perpendicular directions.

The PXRD pattern of the 25% nanoplate composite shown in Figure 3a displays strong preferred orientation in the perpendicular direction compared to the parallel direction of the pellet. The nanoplates align with their planes perpendicular to the SPS direction, therefore significant enhancement of the (00*l*) peak intensities is observed due to the two-dimensional morphology of the nanoplates in the pellet. There is a small impurity peak observed at ∼29°, which may correspond to a metastable phase that was produced in the SPS process, such as BiSbTe_2_ which is a trigonal phase and diffracts at this angle. With only one unassigned peak, it is impossible to unambiguously assign it to a specific phase. Figure 3b shows the SEM micrograph of the parallel direction, where a layered structure is observed, and Figure 3c shows the SEM micrograph of the perpendicular direction, where some of the hexagonal nanoplate morphology is still maintained. The combination of PXRD and SEM of the two directions gives confirmation that there is strong anisotropy within the consolidated material.

**Figure 4:**
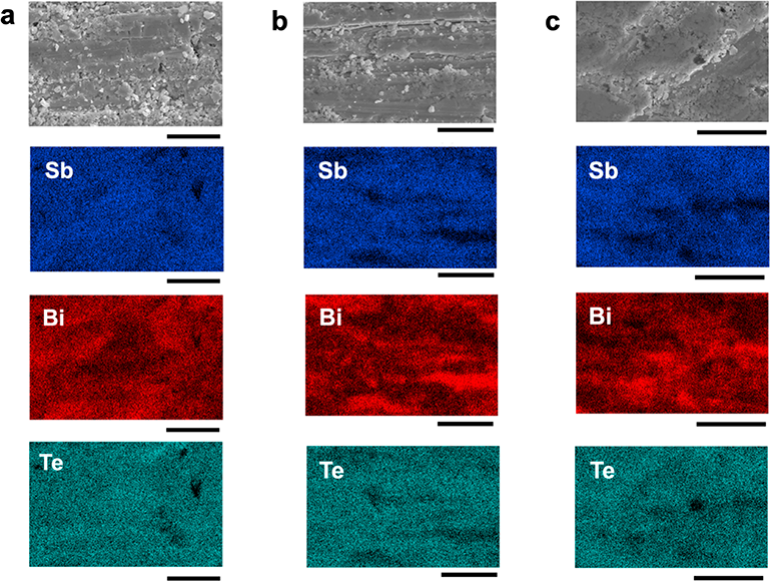
EDX maps of the (a) 15%, (b) 20%, and (c) 25% nanoplate composites, showing increased aggregation as the amount of Bi_2_Te_3_ is increased. All scale bars are 10 μm.

Nanoplate composites of 15%, 20%, and 25% were analyzed by EDX to obtain information about the composition and elemental distribution within the consolidated pellets. The EDX maps of the composites are shown in Figure 4. As the mol % of Bi_2_Te_3_ increases, it appears as though there is more aggregation of the separate phases within the composite. Aggregation of the two phases confirms that the nanoplate interfaces remain intact after the SPS process and there is not a significant amount of diffusion between the two materials.

### 3.3 Thermoelectric Properties

**Figure 5:**
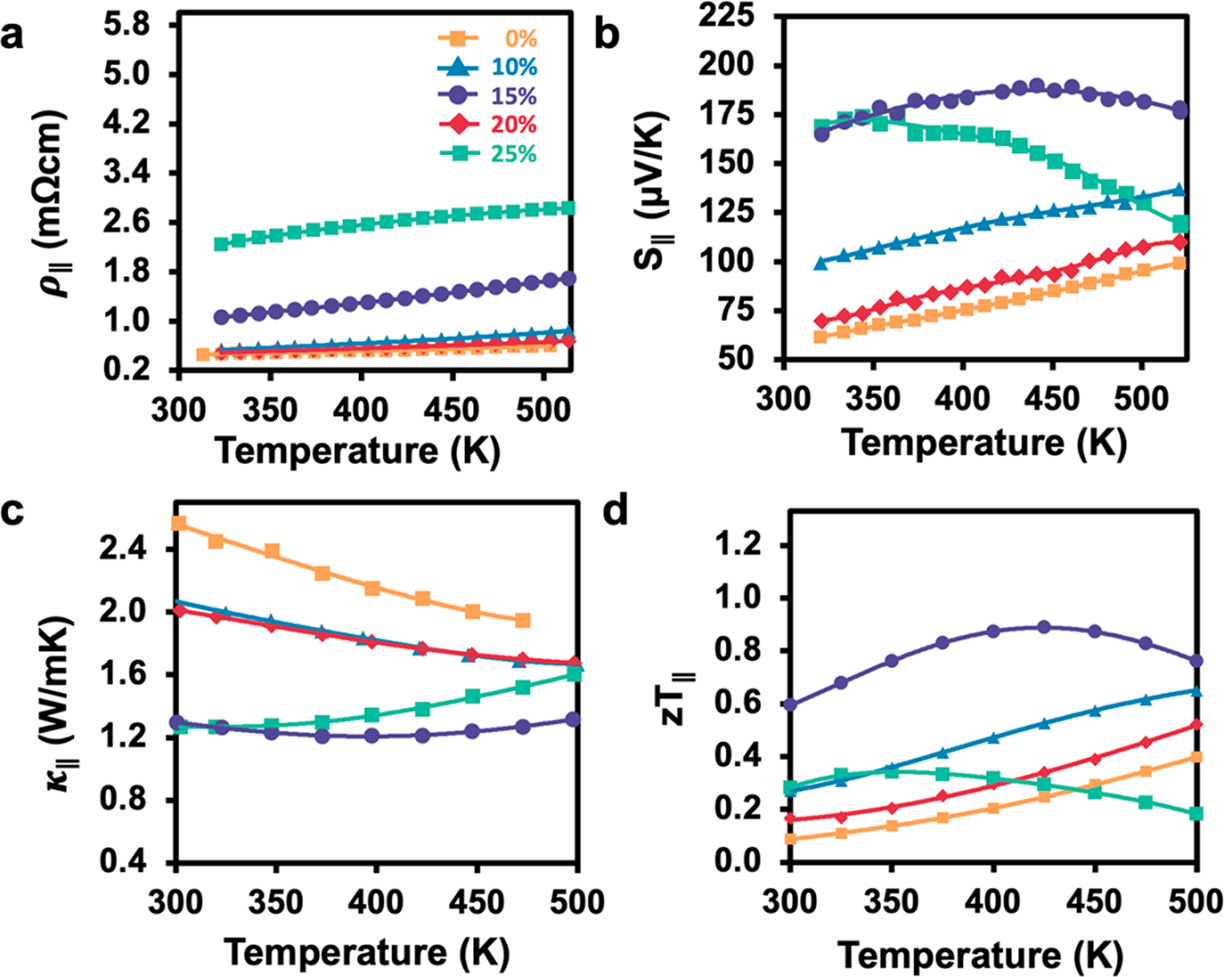
Thermoelectric properties in the parallel direction for nanoplate composites, showing (a) electrical resistivity, (b) Seebeck coefficient, (c) thermal conductivity, and (d) thermoelectric figure of merit, *zT*.

**Figure 6:**
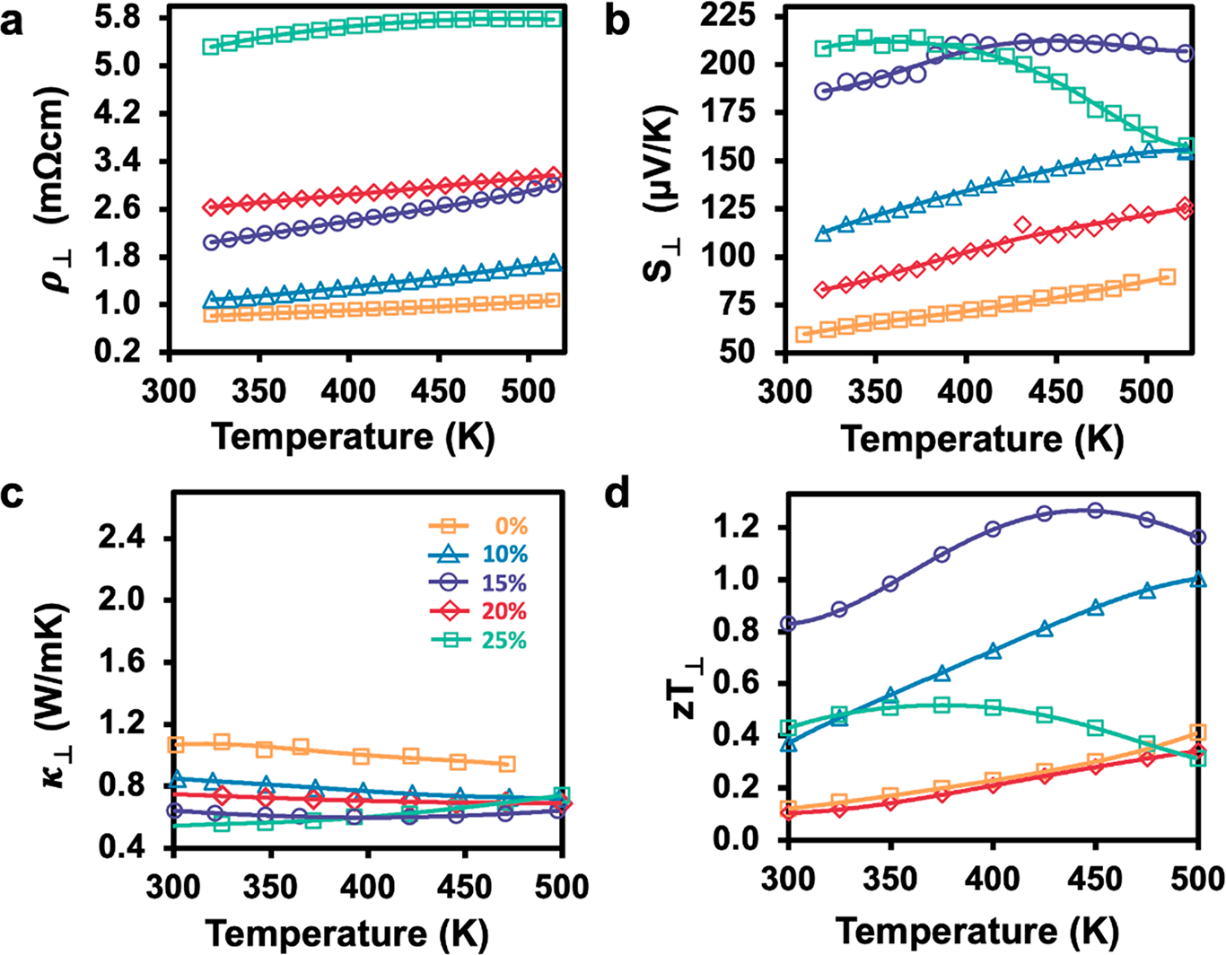
Thermoelectric properties in the perpendicular direction for nanoplate composites, showing (a) electrical resistivity, (b) Seebeck coefficient, (c) thermal conductivity, and (d) thermoelectric figure of merit, *zT*.

The thermoelectric properties are measured for the Sb_2_Te_3_ and Sb_2_Te_3_/Bi_2_Te_3_ nanoplate composites in both parallel and perpendicular directions to elucidate their anisotropy and the impact of the 2D nature of the plates. Figure 5 shows thermoelectric properties measured in the parallel (∥) direction and Figure 6 shows thermoelectric properties measured in the perpendicular (⊥) direction. In the parallel direction, there should be fewer interfaces consistent with the dimensions of the nanoplates, with Sb_2_Te_3_ nanoplates being significantly larger in that dimension. All the as-synthesized Sb_2_Te_3_ and Sb_2_Te_3_/Bi_2_Te_3_ nanoplate composite samples are p-type, as indicated by the positive Seebeck coefficients in Figures 5b and 6b. As-synthesized Bi_2_Te_3_ nanoplate samples are n-type, with negative Seebeck coefficients, which can be seen in Supporting Information, Figure S4. These majority carrier types are consistent with the previously reported antisite defects that Sb_2_Te_3_ and Bi_2_Te_3_ are susceptible to having.^45,46^

The parallel direction resistivity is shown for the nanoplate materials in Figure 5a. Sb_2_Te_3_ nanoplates have the lowest resistivity while the composites increase with Bi_2_Te_3_ nanoplate content. Although, it was observed that the 20% composite has a low resistivity in the parallel direction. All compositions behave as degenerate semiconductors, showing increasing resistivity as temperature increases. At 25% Bi_2_Te_3_, the resistivity begins to flatten, which indicates the onset of bipolar conduction.

The parallel direction Seebeck values reach a maximum of 190 μV/K at 15% Bi_2_Te_3_, as shown in Figure 5b. Sb_2_Te_3_ has a relatively small Seebeck coefficient, which increases with Bi_2_Te_3_ content up to 15%. After 15% Bi_2_Te_3_, the Seebeck coefficient decreases drastically. At 25% Bi_2_Te_3_, there is a bend-over in Seebeck with increasing temperature, which may be attributed to bipolar conduction from significantly high Bi_2_Te_3_ content. The very low Seebeck values for 20% Bi_2_Te_3_ are largely due to the very high carrier concentration, *n*_H_, shown below.

The thermal conductivity for all nanoplate composites in the parallel direction is lowered by the introduction of Bi_2_Te_3_ nanoplates into the Sb_2_Te_3_ nanoplate matrix as is shown in Figure 5c. Bi_2_Te_3_ has a lower thermal conductivity than Sb_2_Te_3_ (Supporting Information, Figure S4) and therefore leads to lower thermal conductivity in the composites. Although, there is an increase at higher temperatures for 25% Bi_2_Te_3_ which we attribute to the onset of bipolar conduction. Again, there is a deviation with 20% Bi_2_Te_3_ content, which has a considerably higher thermal conductivity than 15% and 25% Bi_2_Te_3_ content. This higher thermal conductivity is attributed to the electronic thermal conductivity (*κ*_e_) is dominant in the thermal transport, due to its high carrier concentration, *n*_H_.

All nanoplate composites, except 25%, have an enhanced *zT* in the parallel direction with respect to pure Sb_2_Te_3_ nanoplates, as shown in Figure 5d. For 25% Bi_2_Te_3_ the *zT* is deteriorated by high resistivity and bipolar conduction at higher temperatures. In the parallel direction, the highest thermoelectric performance is attained at 15% Bi_2_Te_3_ nanoplate content, reaching a *zT* of 0.89 at 425 K.

In the perpendicular direction, ρ increases monotonically with increasing Bi_2_Te_3_ content and is consistently higher than in the parallel direction as illustrated in Figure 6a. The higher resistivity is a consequence of charge carrier scattering at the nanoplate interfaces because there are a significantly larger number of interfaces in this direction. To further probe the effect of interfaces on the electrical transport in the nanoplate composites, the temperature dependent Hall carrier mobility (μ_H_) was measured, discussed below, and exhibits significant reduction in the perpendicular direction.

All compositions exhibit significantly higher Seebeck coefficient in the perpendicular direction, with the highest reaching 210 μV/K in the 15% Bi_2_Te_3_ nanoplate composite, shown in Figure 6b. In order to elucidate the mechanism of the enhanced Seebeck values, which is inversely related to *n*_H_ but directly related to effective mass (m*), we investigated m* as a function of composition. It is known that energy carrier filtering will cause an increase to m* as charge carriers encounter high potential barriers upon transport.^47^ Values for m* were calculated for all composites at 323 K using the Seebeck coefficient and Hall carrier concentration, as previously described by Snyder et al.^48^ It is observed that m*, shown in Table 1, is higher for all composites in the perpendicular direction. Although this relatively simple model is most applicable to doped semiconductors of a single-phase material, it also encapsulates the effective mass behavior of modulated materials. We confirm that the enhanced Seebeck coefficient is due to energy carrier filtering in the nanoplate composite because of the higher value of m* in the perpendicular direction.

**Table 1:** Effective Mass (m*) for all Nanoplate Composites Calculated at 323 K

Composition (% Bi_2_Te_3_)	m*_∥_ (m_e_)	m*_⊥_ (m_e_)
10	1.04	1.25
15	0.97	1.20
20	1.21	2.06
25	0.61	0.96

In the perpendicular direction, the thermal conductivity is significantly lower than the parallel direction for all nanoplate composites, shown in Figure 6c. Compared to the thermal conductivity of the pure Sb_2_Te_3_ nanoplates, which is ∼1.06 W/mK, the composites have lower values. Reduction in thermal conductivity can be attributed to the increase in phonon scattering at the nanoplate interfaces. As phonons traverse the composite materials, they encounter lattice mismatch at the heterojunction interfaces, causing a wide range of phonon frequencies to be scattered. At 25% Bi_2_Te_3_, thermal conductivity increases with temperature, attributed to bipolar conduction. For all compositions, lattice thermal conductivity, *κ*_l_, was calculated and displays extremely low values (Supporting Information, Figure S5). The pure Sb_2_Te_3_ nanoplates have the lowest *κ*_l_, approaching negative values, which is a problem that originates from the estimation of the Lorenz number. These ultralow and negative values can arise from inaccuracies in the calculation of the Lorenz number for the degenerate semiconductor limit. It is known that these discrepancies in Lorenz number can reach up to 40% at the degenerate limit.^49^ Sb_2_Te_3_ nanoplates have the lowest Seebeck coefficient and highest carrier concentration, making the calculation of the Lorenz number inaccurate. Although *κ*_l_ is unrealistically low employing this analysis, by subtracting *κ*_e_, the bipolar component of the thermal conductivity can be clearly extrapolated and is seen in 15% and 25% nanoplate composites.

Similarly to the parallel direction, all nanoplate composites except 25% Bi_2_Te_3_ have an enhanced *zT* in the perpendicular direction with respect to pure Sb_2_Te_3_ nanoplates, as shown in Figure 6d. The *zT* values are also higher for all compositions in this direction. The enhanced *zT* values are attributed to a significant reduction in thermal conductivity and a large increase in Seebeck for the perpendicular direction. For 25% Bi_2_Te_3_, the *zT* again exhibits a decline at higher temperatures due to high resistivity and bipolar conduction. In the perpendicular direction, the highest thermoelectric performance is attained at 15% Bi_2_Te_3_ nanoplate content, reaching a *zT* ∼ 1.26 at 450 K. The *zT* continues to increase over the entire temperature range for 0%, 10%, and 20% composites, suggesting that *zT* may be further optimized at higher temperatures for these compositions.

**Figure 7:**
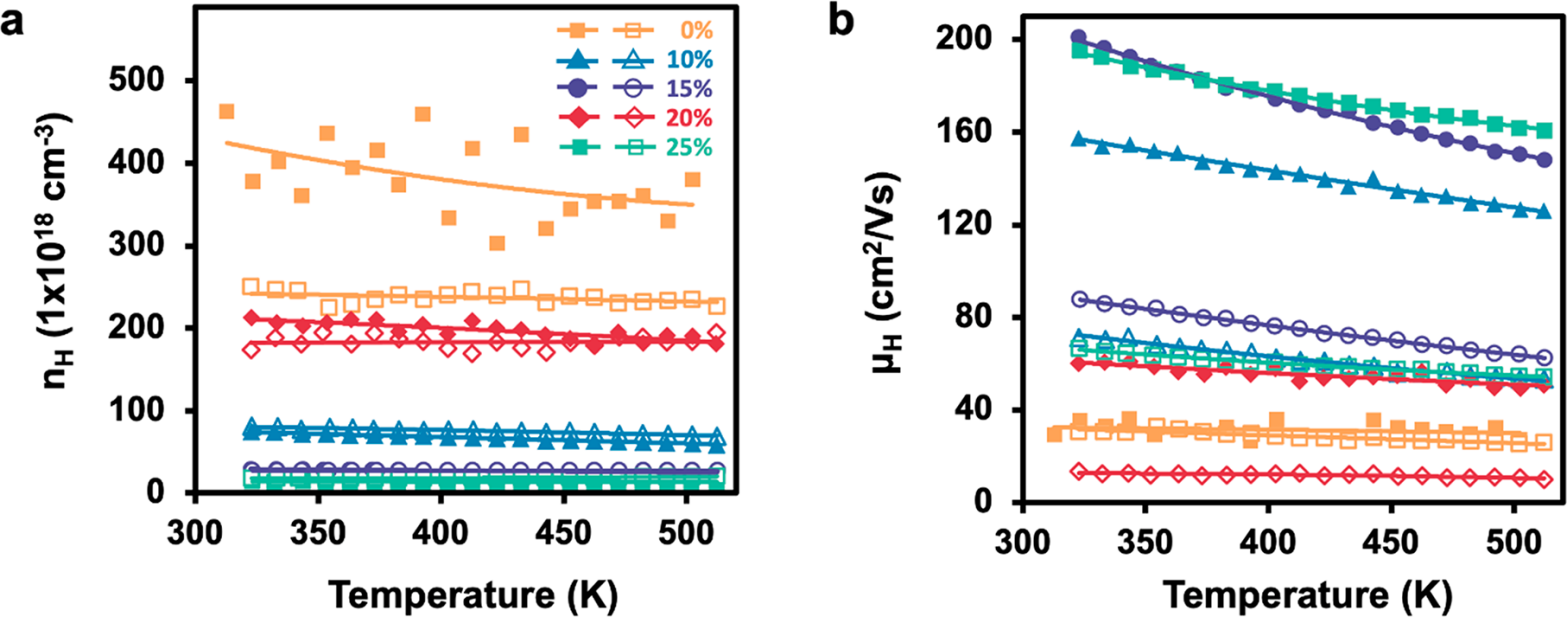
Temperature-dependent transport properties for all composites. Closed markers represent the parallel direction and open markers represents the perpendicular direction for (a) Hall carrier concentration, *n*_H_, and (b) carrier mobility, μ_H_.

As the amount of Bi_2_Te_3_ is increased, the carrier concentration, *n*_H_, generally decreases, with the exception of the 20% composite as shown in Figure 7a. The pure Sb_2_Te_3_ nanoplates have the highest *n*_H_ at ∼4 × 10^20^ cm^–3^ and the 25% composite has the lowest at ∼1.8 × 10^19^ cm^–3^. The 20% composite exhibits a high *n*_*H*_ of ∼2 × 10^20^ cm^–3^ which is close to that of the pure Sb_2_Te_3_ nanoplates. The pure Bi_2_Te_3_ nanoplates have a lower carrier concentration of ∼2.5 × 10^19^ (Supporting Information, Figure S6). The carrier concentration, *n*_H_, shows little dependence on the direction for all composites, further corroborating the negligible effect it has on the Seebeck enhancement with direction.

The *μ*_H_, shown in Figure 7b, monotonically increases with Bi_2_Te_3_ content, although the 20% composite deviates from the trend with very low *μ*_H_. In multiphase composite materials, it is known that the phase with higher *n*_H_ can donate carriers to the phase with lower *n*_H_, which is referred to as modulated doping.^50^ This effect lowers the overall *n*_H_ of the composite materials with respect to the pure Sb_2_Te_3_ nanoplates. At higher Bi_2_Te_3_ content the nanoplates begin to aggregate as shown in Figure 4 and thus do not donate carriers as readily, leading to a higher concentration of holes. This is also reflected in *μ*_H_, which increases with decreasing *n*_H_, due to less ionized impurity scattering. This confirms that the carrier concentration, *n*_H_ was successfully modulated and mobility, *μ*_H_ was greatly enhanced with Bi_2_Te_3_ nanoplate content.

## 4 Conclusion

The phase-pure colloidal synthesis of hexagonal Sb_2_Te_3_ and Bi_2_Te_3_ 2D nanoplates as well as their thermoelectric properties as a function direction for a systematic composite of Bi_2_Te_3_ in Sb_2_Te_3_ are reported. Nanoplates were synthesized via a polyol process and characterized by PXRD, SEM, TEM, SAED, and AFM analysis. The nanoplates are single-crystal nanoplates with sharp hexagonal facets. The Sb_2_Te_3_ and Bi_2_Te_3_ nanoplates vary drastically from one another in their lateral and vertical dimensions.

The thermoelectric properties of the consolidated nanomaterials show strong anisotropy due to the preferred orientation of the nanoplates. This was confirmed by PXRD and SEM analysis of the cross sections of the composite materials. The preferred orientation indicated by the nanoplate (00*l*) indices is observed in the perpendicular direction of the PXRD and layered morphology is seen in SEM cross section micrographs. These findings corroborate the anisotropy of the nanoplates within the composites.

The electrical resistivity generally increases with Bi_2_Te_3_ content, which is due to lowered carrier concentration from modulated doping. The Seebeck values are enhanced in the perpendicular direction for all compositions, reaching a maximum value of 210 μV/K in the 15% composite, an effect we attribute to energy carrier filtering. There is a significant reduction in the thermal conductivity. in the perpendicular direction for all nanoplate compositions.

The thermoelectric performance is increased drastically for all composites, except the highest Bi_2_Te_3_ composite which suffers deterioration of *zT* at the highest temperatures due to bipolar conduction. All *zT* values are higher in the perpendicular direction, due to increased Seebeck coefficients and significantly lowered thermal conductivity. The highest performance, with *zT* ∼ 1.26, is achieved at 15% Bi_2_Te_3_ in the perpendicular direction at 450 K. It is likely that further optimization may be attained for other compositions, such as 10% Bi_2_Te_3_ nanoplate content, at higher temperatures.

## Supporting Information Available

SEM micrograph and PXRD pattern of Sb_2_Te_3_ nanoplates with Te nanorod impurity; SEM micrograph of Bi_2_Te_3_ nanoplates with Te nanorod impurity and nanopore morphology; densities of pressed pellets measured by Archimedes’ method; thermoelectric properties of 2D Bi_2_Te_3_ nanoplate pellet; lattice thermal conductivity of nanoplate composites; electronic carrier concentration and mobility of 2D Bi_2_Te_3_ nanoplate pellet (PDF)
