# Supplementary material for: High Thermoelectric Performance in 2D Sb2Te3 and Bi2Te3 Nanoplate Composites Enabled by Energy Carrier Filtering and Low Thermal Conductivity
Source: ACS Appl Electron Mater. 2023 Jun 5;6(5):2816–25. doi: 10.1021/acsaelm.3c00385 (PMC11137805; doi:10.1021/acsaelm.3c00385)
Supplement: Supplementary file 1 — el3c00385_si_001.pdf [file el3c00385_si_001.pdf]

Supporting Information:

High Thermoelectric Performance in 2D Sb_2Te_3 and Bi_2Te_3 Nanoplate Composites Enabled by Energy Carrier Filtering and Low Thermal Conductivity

Tanner Q. Kimberly,[1] Kamil M. Ciesielski,[2] Xiao Qi,[3] Eric S. Toberer,[2] and Susan M. Kauzlarich*[1]

[1]Department of Chemistry, University of California, One Shields Avenue, Davis, California 95616, United States

[2]Department of Physics, Colorado School of Mines, 1523 Illinois Street, Golden, Colorado 80401, United States

[3]The Molecular Foundry, Lawrence Berkeley National Lab, Berkeley, California 94720, United States

Corresponding author email: smkauzlarich@ucdavis.edu

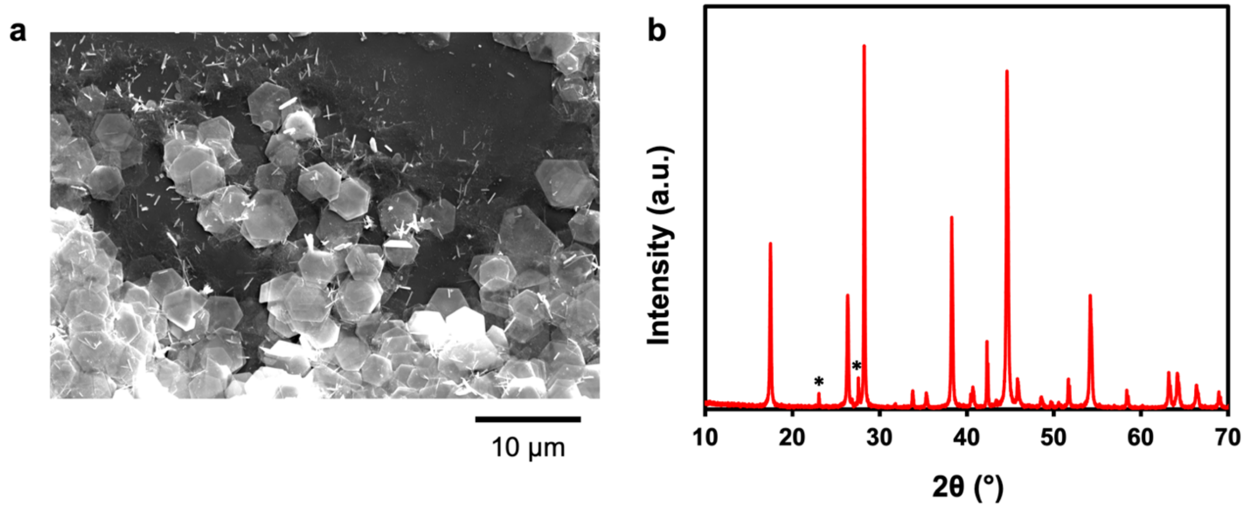

Figure S1. SEM micrograph (a) and PXRD pattern (b) of Sb_2Te_3 nanoplates with Te nanorod impurity. The asterisks in the PXRD pattern corresponds to elemental Te.

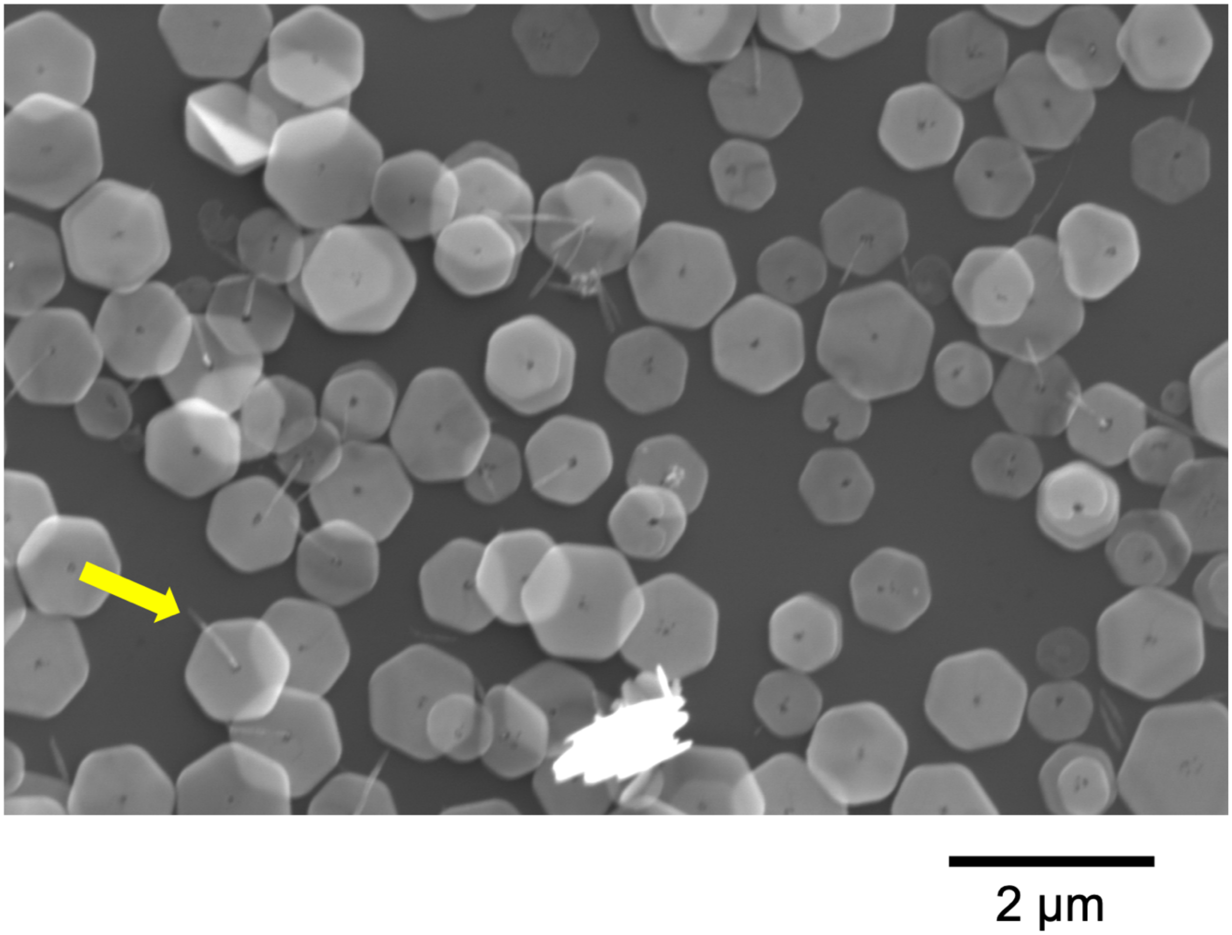

Figure S2. SEM micrograph of Bi_2Te_3 nanoplates with single nanopore and Te nanorod impurity. Yellow arrow pointing to Te nanorod impurity.

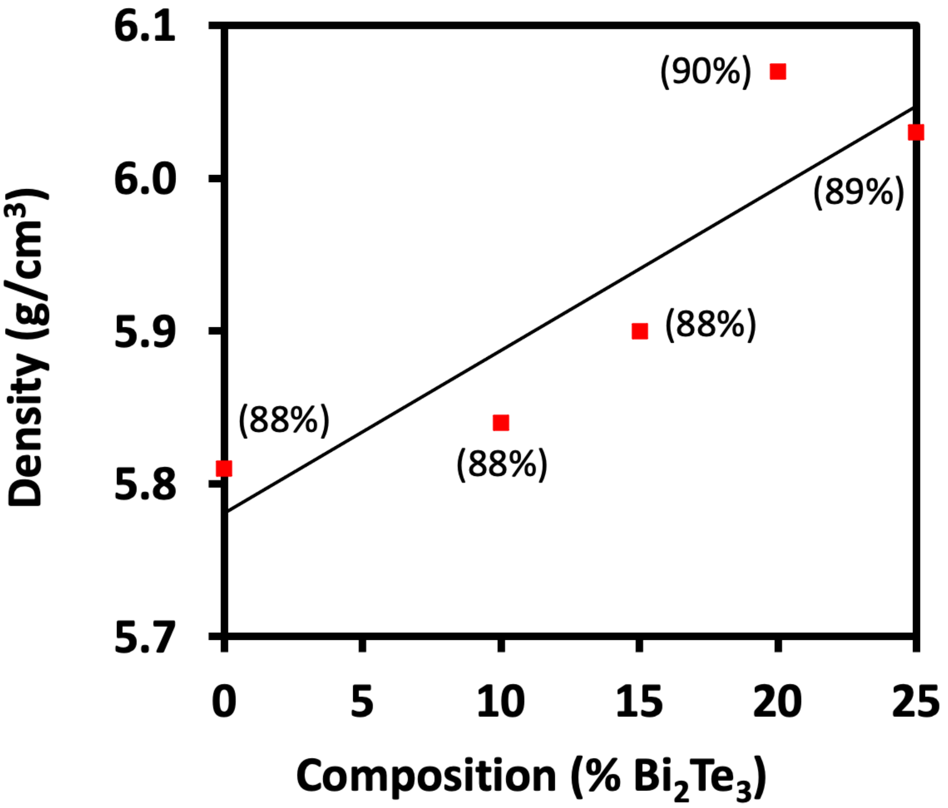

Figure S3. Plot of all nanoplate sample densities as a function of Bi_2Te_3 nanoplate composition (mole %) measured by the Archimedes principle.

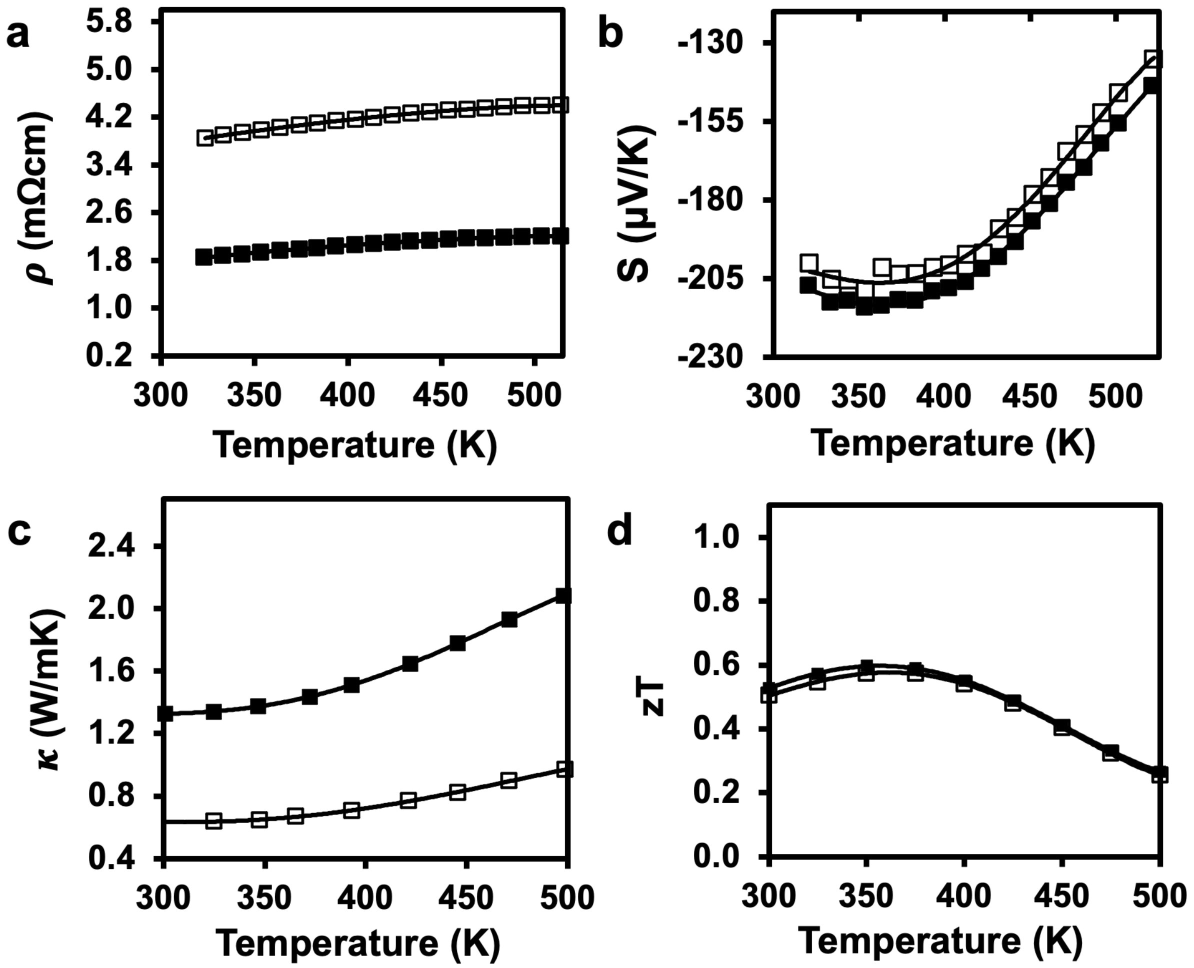

Figure S4. Thermoelectric properties of Bi_2Te_3 nanoplates showing (a) electrical resistivity, (b) Seebeck coefficient, (c) thermal conductivity, and (d) zT. Closed markers represent the parallel direction and open markers represent the perpendicular direction measurements.

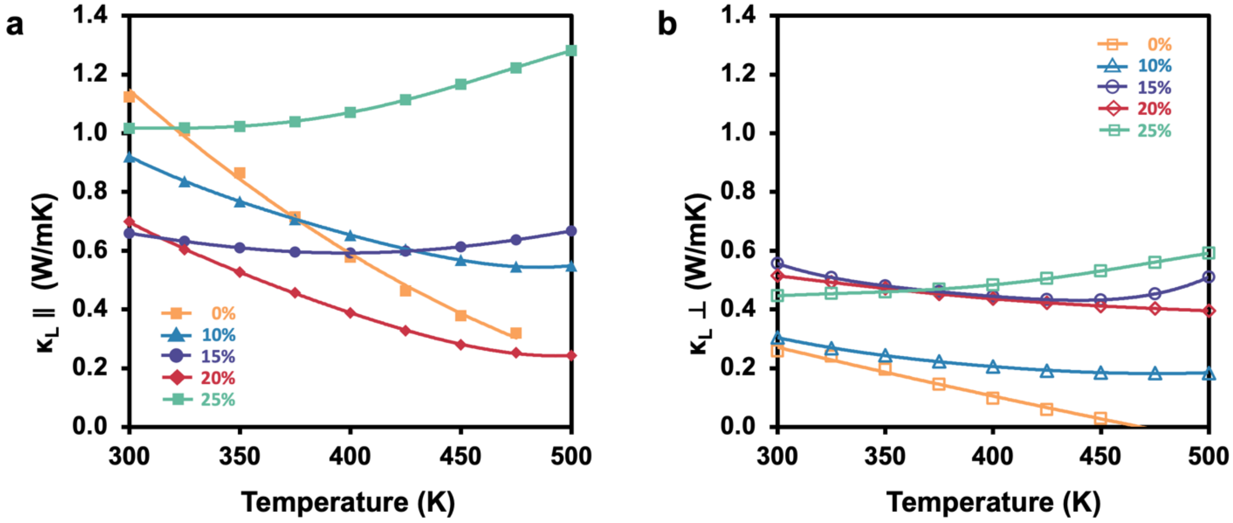

Figure S5. Lattice thermal conductivity for composites calculated for the (a) parallel and (b) perpendicular directions.

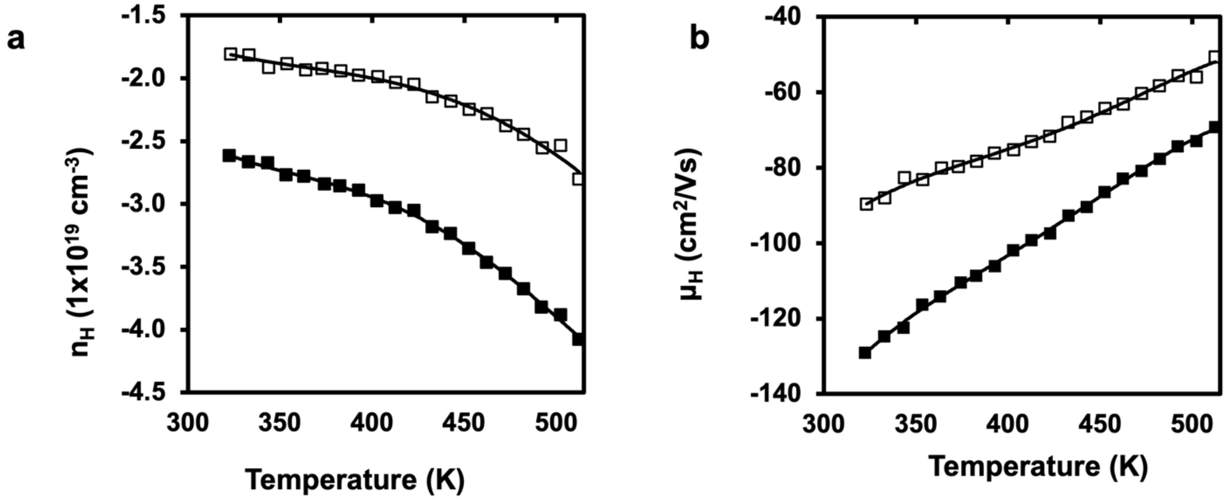

Figure S6. (a) Carrier concentration and (b) mobility of Bi_2Te_3 nanoplates. Closed markers represent the parallel direction and open markers represent the perpendicular direction measurements.
